# Notes and News

**Published:** 1891-04-04

**Authors:** 


### Notes and News.

Collected and Communicated.

A successful First-Aid Class has been instructed by Dr. M. R. Fairer and Dr. B. Walker, at Kirkby-Stephen, Westmoreland. The pupils were all railway employées and were examined by Dr. Altham, of Penrith. At the ceremony of distribution of certificates by Rev. H. A. Feilden, M.A., last week, practical demonstrations of the men's skill were given on the platform which created much amusement. The teachers were afterwards presented with a handsome marble timepiece each, and this week are to be entertained to supper in the Oddfellows' Hall.

The annual general meeting of the Governors of University College Hospital was held on the 25th ult., in the board-room of the institution, Gower Street, Mr. Augustus Provost presiding. Seeing that the year 1890 closed with a debt of £14,147 to bankers and tradesmen new contributions to a large extent are really needed for this old established charity. The income, owing to the partial failure of legacies, is less during the year, and the building fund had unfortunately remained stationary. Additional contributions to the latter fund in order that the hospital may be rebuilt upon a plan more in accordance with the claims of modern medical and surgical treatment were earnestly appealed for in the report.

It appears that the outbreak of diphtheria in Paris might not have occurred but for the want of thought shown by the mother of a little girl who was ill with the disease in the Children's Hospital. After visiting her child she, unaware of the imprudence she was committing, went to take news of the child's health to her schoolfellows and the nuns who taught them. The result of this was that twelve cases occurred in the school, of which five were fatal, and the numbers of children who fell ill at home was very great. Most of them were taken to hospitals in omnibuses by their parents. There seems to be no police regulation to prevent the bodies of those dying of infectious diseases being kept in the vaults of churches pending transportation to provincial or foreign cemeteries.

The annual general meeting of the subscribers to University College Hospital was held on March 25th, Mr. Augustus Prevost, Treasurer and Chairman of the Committee, presiding. The report showed that the old-standing debt to bankers still remains at about £14,000, and although the subscription and donation lists show some improvement, they are still far from sufficient to meet the growing requirements of the hospital. The People's Contribution Fund has again contributed £500, making a total, since the establishment of the fund in 1878, of £4,920. A special appeal is made on behalf of the rebuilding fund, in order to place the institution more in accordance with modern medical and surgical treatment. The report having been adopted, and some new members placed on the Committee, the meeting terminated.

The usual general meeting of the Eye, Ear, and Throat Hospital for Shropshire and Wales was held on March 4th. The surgical report showed that 1,855 new cases had been treated, and 390 patients have been admitted into the hospital; 234 major operations had been performed, including 49 for cataract, 27 for squint, 28 iridectomies, and 15 enucleation. Of the 49 operations for cataract 48 were successful. The financial position of the hospital is satisfactory. For the last two years a private ward has been opened for the reception of one patient at a time. This has been a complete success.

A new wing has been added to the Firs Home at Bournemouth, which adds considerably to the usefulness of the institution. For some years the Home has been worked under great structural difficulties, which are now removed, and, by means of a new heating apparatus, the altered building is made altogether warmer and more comfortable than it has ever been before. Although the Home was closed for alterations for four months, 1890 has been an exceptionally heavy year, every patient having required very special solicitude and attendance. The Firs Home is not a local institution, as patients are received from parts of England, Scotland, and Wales.

The *Standard* understands that the inquiry into the sanitary condition of St. Bartholomew's Hospital, resolved upon by the Commissioners of Sewers, will begin as soon as the Sanitary Committee meets again after the Easter holidays. The proceedings will be private, as is usual with committees of this body, but the report will be made public. There is great reason to doubt whether the Commissioners have any legal power to interfere with the affairs of the hospital, but it is not expected that the authorities of the institution will resist inquiry. The alterations contemplated would not entail great cost, but they would involve the closing of the hospital.

The Hospitals Association has just established, with the permission of the Mercer's Company, their fiftieth street ambulance station, in the portico of the Royal Exchange. The association is now largely extending its organisation, and the Committee have under consideration the establishment of twenty-seven additional stations suggested by the Chief Commissioner of the Metropolitan Police on the recommendation of the superintendents of the different divisions. These stations are in various quarters of the metropolis, but mainly in the East-end, in which district the association's organisation is as yet least complete. The work of equipping these stations will now be vigorously pushed forward, and its completion will see the whole metropolis furnished for the first time with a complete street ambulance service.

Field-Marshal Sir F. Haines presided over the annual meeting of the governors and friends of the Hospital for Diseases of the Throat, Golden Square. Mr. W. T. Sharpe read the 28th annual report, which stated that there had been an increase of patients last year under every head. The in-patients numbered 519, the out-patients 6,855, and the attendances 38,870. The Committee have purchased two houses adjoining the hospital for £3,700, for the purpose of enlarging the institution, but it is impossible to take any steps to utilise that space until a sum of at least £3,000 has been provided, and the Committee make an earnest appeal for that amount. A separate ward for children is greatly needed. On the motion of the Chairman, the report was adopted, and the proceedings terminated with the usual votes of thanks.

